# Enhancement of the Antioxidant Effect of Natural Products on the Proliferation of Caco-2 Cells Produced by Fish Protein Hydrolysates and Collagen

**DOI:** 10.3390/ijms24076871

**Published:** 2023-04-06

**Authors:** Mercedes Taroncher, Yelko Rodríguez-Carrasco, Francisco J. Barba, María-José Ruiz

**Affiliations:** Laboratory of Food Chemistry and Toxicology, Faculty of Pharmacy, University of Valencia, 46100 Valencia, Spain

**Keywords:** hydrolyzates, collagen, bioaccessibility, antioxidant, minerals, metals

## Abstract

A large amount of fish side streams are produced each year, promoting huge economic and environmental problems. In order to address this issue, a potential alternative is to isolate the high-added-value compounds with beneficial properties on human health. The objectives of this study were to determine the effect of hydrolyzed fish protein and collagen samples on cell proliferation, as well as to determine the specific influence of minerals and metals on this effect and whether dietary antioxidants can enhance cell proliferation. The results of hydrolyzed fish protein and collagen samples showed negative effects on Caco-2 cell proliferation at the highest concentrations tested. Moreover, the pre-treatment of these hydrolyzates with vitamin C and E, quercetin and resveratrol increased the proliferation of bioaccessible fractions of hydrolyzated fish protein and collagen samples compared to the bioaccessible fractions without pre-treatment. The highest mineral concentrations were found for P, Ca and Mg. The metals found in the pure hydrolyzates were As, Cd, Hg and Pb; however, they appeared at almost undetectable levels in bioavailable fractions. It can be concluded that the consumption of hydrolyzates of fish by-products is an interesting strategy for complying with EFSA recommendations regarding fish consumption while at the same time reducing fish waste.

## 1. Introduction

Marine organisms are reported to produce a variety of highly biologically active high-added-value compounds as they are forced to live in a complex environment which is exposed to extreme conditions of salinity, pressure, temperature and illumination [1]. Fish is a rich source of protein, with levels ranging from 10 to 25% and differing according to the species; this protein is mainly located in fish muscle [2]. Fish protein hydrolyzates are desirable functional food ingredients due to their natural availability, low-cost extraction methods and their ability to produce beneficial effects on human health by exhibiting antioxidant, anti-inflammatory, anti-proliferative, anti-hypertensive and cardio-protective bioactivities [3]. For instance, marine collagen has shown promising activities such as antioxidant, wound healing, anti-aging, adipogenic differentiation inhibition, and anti-freezing activity, among others [4]. Salmon consumption has tripled since the 1980s, mainly because it is considered a healthy food due to its contents of polyunsaturated fatty acids, high-biological-value quality proteins, vitamins, and minerals [5,6]. In fact, increased growth in the salmon aquaculture sector has been observed worldwide. This can be justified by taking into account the high health perception of consumers regarding salmon consumption. In Europe, Atlantic salmon (*Salmo salar*) is currently the most important farmed specie in volume and value [7]. Since salmon has a great fillet yield, it is one of the most highly processed kinds of fish [8]. Leftovers derived from salmon processing include heads (containing the gills), trimmings (containing muscle, bone and skin), mince, frames and viscera (liver, kidney and roe) [9]. As a result, 50% of complete fresh salmon has been estimated to correspond to side-stream materials [10]. Without proper treatment, contamination and disposal problems could occur [8]. Over the last two decades, those side streams have been converted to an array of products, including collagen, gelatin, oils and hydrolyzates. Simultaneously, the production of protein hydrolyzates is a cheaper way to reduce environmental problems while obtaining some high-added-value products. Therefore, salmon side-stream materials could be considered as a promising source of valuable compounds from a European circular economy point of view [11]. Moreover, the opportunities to produce nutritional and functional products from fish side-stream biomasses (such as promising food supplements) are great and its practice would be environmentally responsible, sustainable and resource efficient.

Numerous studies have shown that the consumption of natural products in the form of beverages and diets rich in fruits and vegetables have a cytoprotective effect since many antioxidants may act as free radical scavengers, resulting in an improved endogenous defense system [12,13,14,15,16,17]. Vitamin C (vit C) is an effective scavenger of hydroxyl radicals and a potent antioxidant. Interestingly, it triggers both enzymatic defense reactions (glutathione peroxidase, superoxide dismutase and catalase) and nonenzymatic defense reactions (glutathione and antioxidants from the diet) [18]. Vitamin E (vit E) is a lipid radical chain breaker that scavenges oxygen radicals and alkyl radicals. Due to its antioxidant and anti-inflammatory protective effect, vit E therapy has been utilized for the prevention of inflammatory and aging diseases [19].

On the other hand, quercetin (3, 3′, 4′, 5, 7-pentahydroxyflavone; QUE) and resveratrol (3, 5, 4′-trihydroxystilbene; RSV) are polyphenols both occurring in vegetables in significant amounts. QUE, a naturally occurring dietary flavonol compound, has shown great beneficial effects in different areas relevant to human health. For instance, the beneficial health effects of QUE, including anti-cancer, anti-inflammatory, anti-diabetic, anti-allergic and anti-neurodegenerative properties, have been studied in cell and animal models [13,14,15]. The beneficial properties of QUE have been mainly attributed to its strong antioxidant and chelating capacities. The interest in the study of QUE is mainly due to its wide availability among dietary sources, contributing to approximately 75% of total flavonol intake. RSV is a stilbene abundant in grapes and grape products such as wines and grape juice. It has different biological properties, including neuroprotection, inhibition to the oxidation of low-density lipoproteins and anti-inflammatory, antioxidant, anti-aging, anti-diabetic and antiplatelet properties, thus preventing several human diseases [16]. These potential benefits could lead to an increased consumption of dietary antioxidant supplements by consumers. This approach of consuming fish products supplemented with antioxidants has several benefits, including being cheap, easy to handle and physiologically safe. This may provide a conventional strategy to enhance the beneficial effect of fish side streams [20].

The beneficial effects of hydrolysates have been outlined by Taroncher et al. [21]. Fish and their derived products are considered as a good nutritional source because of their high mineral content. Nevertheless, information in the literature regarding the concentrations of minerals in the fish side-stream products studied in this work is limited. However, the health-related effects of minerals from fish products differ not only according to their content, but also their bioavailability. For this purpose, we carried out a simulated in vitro digestion process, which allows for estimates of bioaccessibility (the total amount of minerals and metals in soluble form released from the solid food matrix that is available for absorption), the preliminary step of bioavailability. On the other hand, farmed fish can be exposed to potentially harmful substances, such as toxic metals from the aquaculture environment [22]. Some metals have been found in several fish side streams, as reported elsewhere [23]. Therefore, assessing the levels of toxic elements is recommended.

In vitro cytotoxicity tests are a part of some alternative methods, whose purpose is the fulfillment of the “3Rs”. They are often applied to preliminary research results prior to in vivo methods due to their cost efficiency and ability to provide fast and reproducible data [2].

Taking into account the above information, the specific aims of this study were to evaluate (i) the cytotoxic effects of hydrolyzed fish protein and collagen; (ii) the ability of antioxidants vit C, vit E, QUE and RSV to increase the beneficial effects produced by the hydrolyzed fish protein and collagen in human colon adenocarcinoma cells (Caco-2); and (iii) the bioaccessibility and bioavailability of the macro- (K, Ca, Mg) and oligoelements (Fe, Zn) and metals provided by hydrolyzed fish protein and collagen.

## 2. Results

### 2.1. In Vitro Cytotoxicity

**Figure 1:**
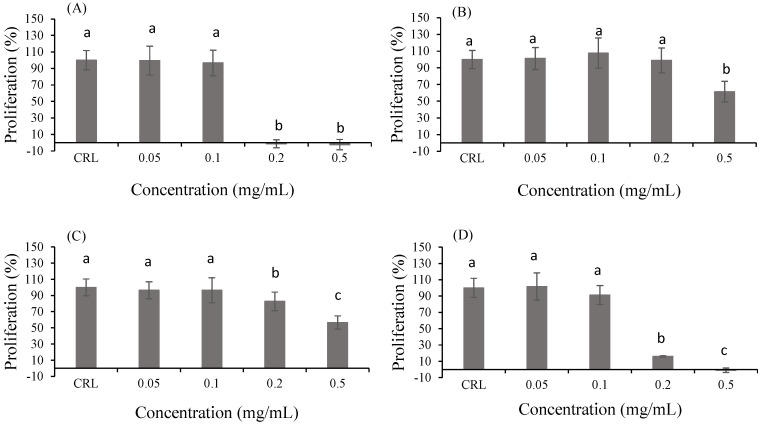
Effect of bioaccessible fractions of product 1 (**A**), product 2 (**B**), product 3 (**C**) and collagen (**D**) on cell proliferation in Caco-2 cells after 24 h of exposure at increasing concentrations from 0.05 to 0.5 mg/mL as assessed by the MTT assay. Values are expressed as mean ± SEM (*n* = 3). Values in the same figure with different superscript letters are significantly different (*p* < 0.05). CRL: control.

Evaluation of the proliferative effect of the bioaccessible fraction of products 1, 2, 3 and collagen in Caco-2 cells was evaluated using an MTT assay over 24 h. The concentration range of the samples selected to study the potential cytotoxic effects on these cells was 0.05, 0.1, 0.2 and 0.5 mg/mL. After 24 h of treatment, no significant effects on mitochondrial function in Caco-2 cells at 0.05 mg/mL and 0.1 mg/mL of bioaccessible fraction were observed for any of the samples studied (Figure 1). However, cytotoxic effects in almost all samples were observed after 24 h of exposure at 0.2 mg/mL and 0.5 mg/mL of bioaccessible fraction. Product 2 at 0.2 mg/mL did not show differences compared to control samples. Cell viability decreased 100% for product 1 and collagen, 38.62% for product 2 and 43.46% for product 3. Product 1 showed the highest decrease in cell proliferation at the 0.5 mg/mL concentration.

### 2.2. Effects of Vitamins and Polyphenols

**Figure 2:**
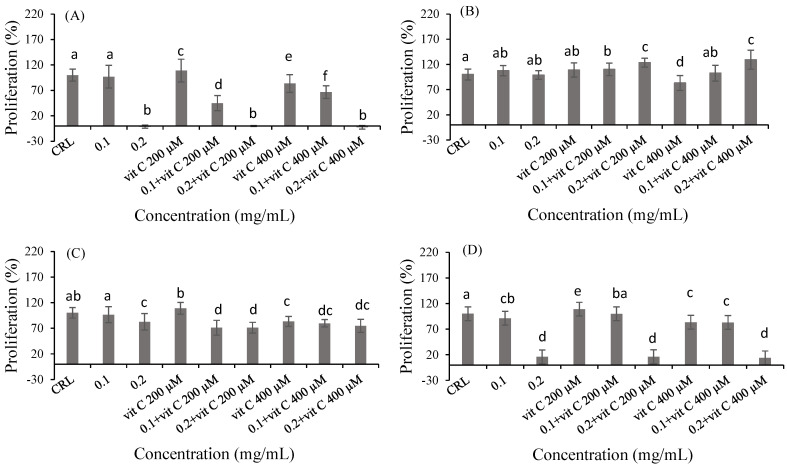
Protective effect of vit C (200 and 400 µM) on the bioaccessible fractions (0.1 and 0.2 mg/mL) of product 1 (**A**), product 2 (**B**), product 3 (**C**) and collagen (**D**) in Caco-2 cells after 24 h of exposure as assessed by the MTT assay. Values are expressed as mean ± SEM (*n* = 3). Values in the same figure with different superscript letters are significantly different (*p <* 0.05). CRL: control.

**Figure 3:**
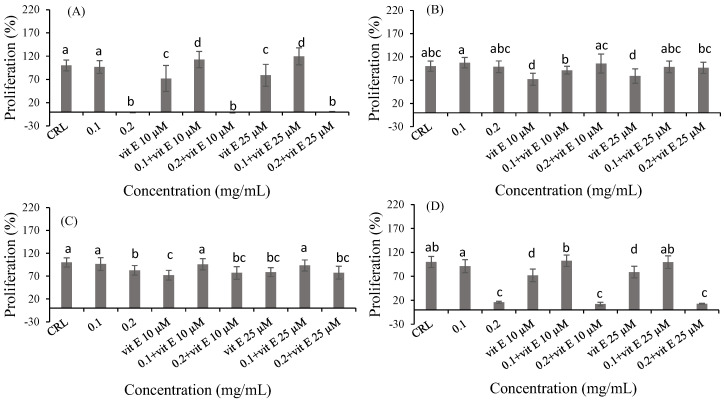
Protective effect of vit E (10 and 25 µM) on the bioaccessible fractions (0.1 and 0.2 mg/mL) of product 1 (**A**), product 2 (**B**), product 3 (**C**) and collagen (**D**) in Caco-2 cells after 24 h of exposure as assessed by the MTT assay. Values are expressed as mean ± SEM (*n* = 3). Values in the same figure with different superscript letters are significantly different (*p <* 0.05). CRL: control.

To determine if vitamins and polyphenols could increase the cell proliferation induced by fish products, two different concentrations of bioaccessible fraction were selected (0.1 mg/mL (no cytotoxic) and 0.2 mg/mL (cytotoxic)). The effect of vit C, vit E, RSV and QUE on cell proliferation was evaluated by the MTT assay. Significant differences were observed after vit C pre-treatment in 0.1 and 0.2 mg/mL concentrations of bioaccessible fractions of product 2 and collagen, as shown in Figure 2. In product 2 (Figure 2B), there was an increase in cell proliferation for 200 µM vit C (24.92%) and 400 µM vit C (30.88%) at 0.2 mg/mL of bioaccessible fraction compared to product 2 without pre-treatment. Finally, in collagen (Figure 2D), an increase of 9.46% in cell proliferation for 200 µM vit C at 0.1 mg/mL of bioaccessible fraction was observed compared to 0.1 mg/mL of bioaccessible fraction without pre-treatment. Regarding vit E pre-treatment (Figure 3), an increase in cell viability after pre-treatment with 10 µM vit E (16.65% and 56.80%) and 25 µM vit E (23.89% and 52.00%) at 0.1 mg/mL of the bioaccessible fraction of product 1 was observed compared to 0.1 mg/mL of the bioaccessible fraction of product 1 without pre-treatment (Figure 3A). In product 2 (Figure 3B), an increase in cell proliferation for 25 µM vit E at 0.1 and 0.2 mg/mL of bioaccessible fraction (24.75% and 22.59%, respectively) was observed compared to 25 µM vit E. In product 3 (Figure 3C), there was an increase in cell proliferation for 10 µM vit E at 0.1 mg/mL of bioaccessible fraction (33.14%) compared to 10 µM vit E. Additionally, in 0.1 mg/mL of the bioaccessible fraction of collagen (Figure 3D), an increase in cell proliferation of 12.35% and 42.50% was observed after 10 µM vit E pre-treatment with respect to 0.1 mg/mL of the bioaccessible fraction of collagen and vit E 10 µM, respectively. Moreover, an increase of 26.30% in cell proliferation for 25 µM vit E at 0.1 mg/mL of bioaccessible fraction was also observed compared to 25 µM vit E (Figure 3D).

**Figure 4:**
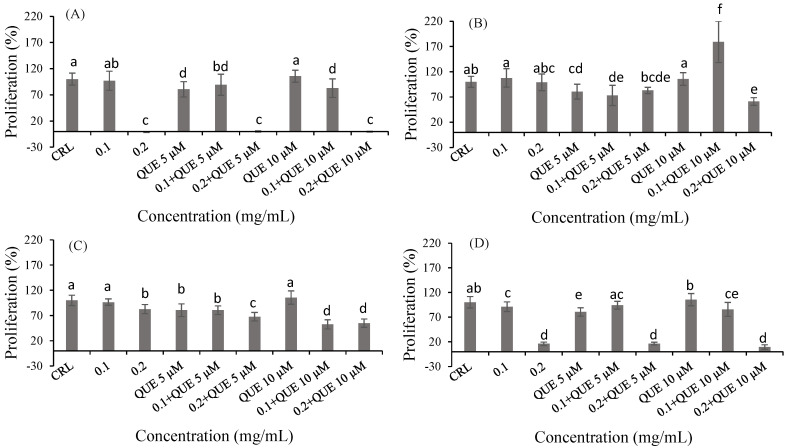
Protective effect of QUE (5 and 10 µM) on the bioaccessible fractions (0.1 and 0.2 mg/mL) of product 1 (**A**), product 2 (**B**), product 3 (**C**) and collagen (**D**) in Caco-2 cells after 24 h of exposure as assessed by the MTT assay. Values are expressed as mean ± SEM (*n* = 3). Values in the same figure with different superscript letters are significantly different (*p <* 0.05). CRL: control; QUE: quercetin.

According to QUE pre-treatment (Figure 4) in product 2 (Figure 4B), an increase in cell proliferation (66.37%) was observed at 0.1 mg/mL of bioaccessible fraction with 10 µM QUE compared to 0.1 mg/mL of bioaccessible fraction alone.

**Figure 5:**
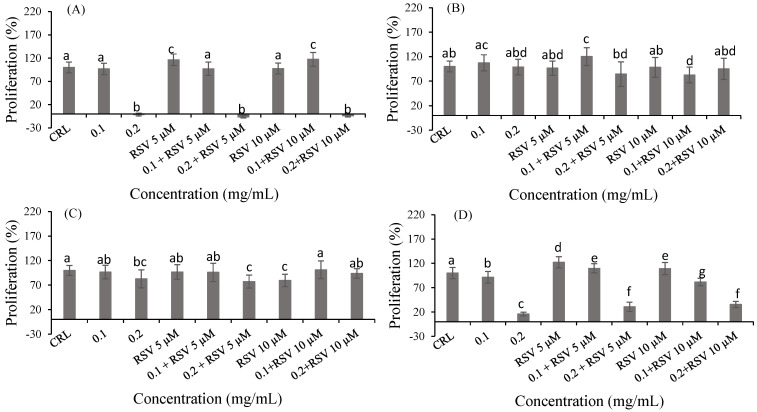
Protective effect of RSV (10 and 25 µM) on the bioaccessible fractions (0.1 and 0.2 mg/mL) of product 1 (**A**), product 2 (**B**), product 3 (**C**) and collagen (**D**) in Caco-2 cells after 24 h of exposure as assessed by the MTT assay. Values are expressed as mean ± SEM (*n* = 3). Values in the same figure with different superscript letters are significantly different (*p <* 0.05). CRL: control; RSV: resveratrol.

Lastly, after RSV pre-treatment in product 1 (Figure 5A), an increase in cell proliferation was observed at 0.1 mg/mL of bioaccessible fraction pre-treated with 10 µM RSV (21.38%) compared to 0.1 mg/mL bioaccessible fraction without pre-treatment. With collagen, an increase of 20.27% was observed at 0.1 mg/mL of bioaccessible fraction with 5 µM RSV compared to 0.1 mg/mL of bioaccessible fraction without pre-treatment. Likewise, an increase of 91.50% and 122.10% was observed at 0.2 mg/mL of the bioaccessible fraction of collagen with 5 and 10 µM RSV, respectively, compared to 0.2 mg/mL of bioaccessible fraction without pre-treatment (Figure 5D).

### 2.3. Content of Minerals in Fish Products

The total content of mineral elements in fish products before and after gastrointestinal digestion is reported in Table 1, Table 2 and Table 3. Before simulated gastrointestinal digestion, the predominant element was P for products 1, 2 and 3 (366.0–565.0 mg/L), while Ca was the most abundant for collagen (633.0 mg/L) samples (Table 1). In samples after simulated gastrointestinal digestion (Table 2), the predominant element was P for product 1 and 3 (46.0–52.6 mg/L), while Mg was the most abundant in product 2 (30.1 mg/L) and Ca was the most abundant in collagen samples (59.1 mg/L). Moreover, P was the predominant (0.1–1.6 mg/L) mineral in the bioavailable fraction of product 1, product 3 and collagen (Table 3).

The second most abundant element in samples before simulated gastrointestinal digestion was Mg (76.0–218.0 mg/L) for all fish products and collagen samples, followed by Ca (50.0–85.8 mg/L) for products 1, 2 and 3 and P (25.5 mg/L) for collagen samples (Table 1). After simulated gastrointestinal digestion (Table 2), the second most abundant element for product 1 was Mg (9.1 mg/L), followed by Ca (7.9 mg/L). Regarding product 2, the second most abundant element was Ca (17.7 mg/L), followed by P (7.4 mg/L). For product 3, the second most abundant elements were Ca (16.7 mg/L) and Mg (16.6 mg/L). Finally, for collagen, the second most abundant element was Mg (7.7 mg/L). The oligoelements Fe, Zn and Se were in the minority in all samples.

A decrease in the amount of macro-elements and micro-elements occurred after the bioavailability process (Table 3). For instance, a 100% decrease was found for Mg, Ca and Fe for all fish samples, while P decreased from 93.73% (collagen) to 100% (product 2), Zn from 97.54% (product 1) to 100% (product 3) and Se from 97.96% (product 2) to 99.48% (product 1, product 3 and collagen).

### 2.4. Content of Metals in Fish Products

The concentration of As, Cd, Hg and Pb in products 1, 2 and 3 and collagen are shown in Table 4 and Table 5. The mean concentration ranges before simulated gastrointestinal digestion, expressed as μg/Kg, were 72.60–1421.00, <0.20–16.50, 0.13–4.50 and 1.10–2.06 for As, Cd, Hg and Pb, respectively (Table 4). For all products before and after the simulated gastrointestinal digestion, the most abundant element was As and the least abundant were Pb and Hg (Table 4 and Table 5).

## 3. Discussion

Fish side streams contain many proteins and other bioactive compounds of high nutritional value. The effective use of these fish side streams contributes to the generation of high-added-value food products for industry. The extracts and fish protein hydrolyzates can have different applications, including use as antimicrobials, antioxidants and dyes to improve the nutritional profile of foods [21]. Most authors have determined that fish protein hydrolyzates and fish collagen are great candidates for the development of dietary supplements because of their numerous benefits. However, peptides of these fish hydrolyzates are often chemically and physically unstable, display rapid clearance, and are quickly degraded by enzymes [24]. Therefore, the bioaccessibility of these protein hydrolyzates should be evaluated. In this study, protein hydrolyzates from salmon, mackerel and blue whiting with the addition of vitamin C and D3 and flounder skin collagen were selected to study proliferative effects in Caco-2 cells. Serial dilutions of each bioaccessible fraction of protein hydrolyzates were chosen to evaluate the effect on cell proliferation of this cell type. According to the MTT assay, the hydrolysates showed cytotoxic effects with increasing concentrations when the cells were exposed to products 1, 2 and 3 and collagen (Figure 1). Most of the studies evaluating the impact of fish protein hydrolyzates on several human cell lines found a decrease in cell viability when they were used at high concentrations. Similar to our results, Marrón-Grijalba et al. reported a decrease in HeLa cervical cancer cell viability to less than 50% when they increased the concentration of *Hypanus dipterurus* spine extract (between 0.1 and 0.2 mg/mL) [25]. These results are in agreement with Rajeshkumar et al., who determined that the *Dasyatis sephen* marine stingray integumentary sheath showed antiproliferative activity against HeLa cells in a concentration-dependent manner [26]. Moreover, Fuochi et al. demonstrated that *D. pastinaca* mucus was toxic to acute leukemia cells (HL60) with an IC_50_ at the concentration of 1 mg/mL [27]. However, as far as we know, no data about the effects of bioaccessible fractions of fish protein hydrolyzates are available in the literature yet, though we can conclude that the trend of decreasing cell proliferation effect with increasing concentrations is the same for pure hydrolyzates.

Product 1, based on hydrolyzed salmon fish protein, increased cell proliferation after the addition of vit E and RSV. Product 2, based on hydrolyzed salmon and mackerel fish protein, increased cell proliferation after addition of vit C and QUE. Furthermore, flounder skin collagen increased cell proliferation when vitamin C, vitamin E and RSV were added. Thus, vitamin C, vitamin E and RSV seem to be the most suitable to consume as antioxidant dietary supplements with the ingestion of protein fish hydrolyzates and fish collagen. The results show that among the studied antioxidant compounds, vitamin C, vitamin E and RSV increased cell proliferation in Caco-2 cells. Therefore, they are considered more suitable for addition to these hydrolyzates if they were to be marketed as a possible bioactive product or food supplement to reduce fish waste generated by the fishing industry. However, since the formulation of products 1 and 2 includes vitamin C, the effect would be further enhanced by supplementing the diet with vit E and RSV, especially as RSV shows cell proliferation increases of up to 91.50% and 122.10% at 0.2 mg/mL of the bioaccessible fraction of collagen at volumes of 5 and 10 µM, respectively.

Similarly, Demircioğlu and Öztürk reported that RSV delayed oxidative processes in fish salami and can be used as a good alternative to vit C [28]. Thus, considering these positive effects, RSV has the potential to be used as an additive in fish products. Wang et al. also studied the benefits of three antioxidants (brown seaweed polyphenols, α-tocopherol (vit E) and ascorbic acid (vit C)) on protein oxidation and the textural properties of fish mince (*Pagrosomus major*) during frozen storage. They found increased protein oxidation during the storage of fish mince, thus promoting the formation of carbonyls, which may cause the quality of seafood to become unacceptable. The three antioxidants proved their effectiveness in retarding protein carbonyl formation, especially vit E. In addition, all three antioxidants improved the texture of fish gel during frozen storage because they showed good activity in terms of the breaking force of the gels during the storage period [29].

Due to the fact that consumers are interested in the mineral content of fish products, the essential minerals Mg, P, Ca, Fe, Zn and Se were determined. These elements are also potent antioxidants in different cell lines [30,31,32,33,34]. No data about the detection of minerals in the bioaccessible fractions of fish hydrolyzates have been studied yet; however, some authors have evaluated the content of minerals in fish extracts. In our samples, before simulated gastrointestinal digestion, we found mineral levels lower than those of other marine fishes. In this line, Chai et al. detected 41.89 ± 0.22 mg/Kg of Zn and 3.71 ± 0.46 mg/Kg of Se in *Benthosema pterotum* [30]. Moreover, Fe levels of 15.49 ± 1.15 mg/Kg and 2.07 ± 0.11 mg/Kg and Zn levels of 7.65 ± 0.46 mg/Kg and 10.36 ± 0.76 mg/Kg were found in the head and mantle of cuttlefish, respectively [35]. Gonzalez et al. determined similar values for micro- (Fe, Zn and Se) and macro-minerals (Mg, P and Ca) in wild and farmed yellow perch fillets. In wild yellow perch fillets, they found values of 4.65 ± 2.07 pmg/Kg, 7.24 ± 0.53 mg/Kg and 1.61 ± 0.15 mg/Kg for Fe, Zn and Se, respectively, while the values for Mg, P and Ca were 220 ± 10 mg/Kg, 1640 ± 40 mg/Kg and 160 ± 20 mg/Kg, respectively. In farmed yellow perch fillets, they found values of 4.90 ± 1.22 mg/Kg, 5.91 ± 1.02 mg/Kg and 1.20 ± 0.13 mg/Kg for Fe, Zn and Se, respectively, while the levels of Mg, P and Ca were 290 ± 10 mg/Kg, 2080 ± 40 mg/Kg and 280 ± 50 mg/Kg, respectively. However, similarly to our results, P was the predominant mineral in wild and farmed yellow perch fillets, followed by Mg [36].

On the other hand, it should be noted that the limits for metals in fish side streams are not currently regulated. Therefore, safety assessment could be based on the limit values established for edible muscles of fish (µg/Kg): 13,500 for As, 50 for Cd, 500 for Hg and 300 for Pb [22,23]. According to this, the levels of metals analyzed in all hydrolysed salmon and collagen evaluated in this study are below the limits set by authorities, so they could be considered safe for consumers.

A study conducted by de la Fuente et al. determined the concentrations of As, Hg, Cd and Pb in the muscle, heads, viscera, skin and tailfins of salmon side streams before simulated gastrointestinal digestion. Regarding As, the results for viscera, skin and tailfins from salmon side streams (461.7, 450.4 and 418.6 µg/Kg, respectively) were similar to those obtained in product 2 of this work (307 µg/L). Similarly, regarding Cd, the values for the heads, tailfins, skin and muscles of salmon side streams (1.1, 10.4, 1.9 and 0.4 µg/Kg, respectively) were very close to the values of 1.2, 16.5, 2.35 and 0.2 µg/L obtained in our study for product 1, 2 and 3 and collagen, respectively [37]. Moreover, for all products before and after the simulated gastrointestinal digestion, the predominant element was As, similar to the study conducted by de la Fuente et al. in which As was determined as the most abundant element for all salmon side streams [11]. However, Pb was not detected in any of the products after simulated gastrointestinal digestion, contrary to the results determined by de la Fuente et al. Moreover, de la Fuente et al., Kalantzi et al. and Kandyliari et al. evaluated the content of As, Hg, Cd and Pb in several fish side streams of sea bass, sea bream and meager [22,23,37,38], with As levels in the viscera (1867–2587 µg/Kg, *w*/*w*) of these fish species being higher than the values obtained in this work (1421–72.6 µg/L). Furthermore, Khristoforova et al. reported accumulations of Hg in the liver in four wild species of Pacific salmon [39]. The results (120–192 µg/Kg, *w*/*w*) were much higher than those found in the present study for all products and collagen (0.1–4.5 µg/Kg). Other authors determined the levels of Cd and Pb in 21 samples of smoked salmon from a Polish market. For Cd, the range of concentrations (4–19.6 µg/Kg *w*/*w*) was very similar to the range that we obtained in this work (0.2–16.5 µg/Kg). However, for Pb, the range of concentrations (10.9–155.9 µg/Kg *w*/*w*) was very different to the range we determined. Nevertheless, both are considered safe for consumers [40].

On the other hand, there is a lack of information in the literature regarding the metal content of the bioavailable fractions of discarded salmon products. The levels of As, Hg, Cd and Pb in the hydrolyzed salmon fish protein and collagen of this study contribute not only to increasing the limited data in the literature about these contaminants in farmed fish, but also provide information about their safety as candidates for value addition and human consumption.

To sum up, product 2 and collagen presented the highest percentage increases in cell proliferation with antioxidant pre-treatments (QUE and RSV), with the predominant minerals being Mg (for product 2) and Ca (for collagen); thus, these may be the most suitable minerals for enhancing cell proliferation. Additionally, product 2 and collagen contained the least amount of metals after simulated gastrointestinal digestion.

## 4. Materials and Methods

### 4.1. Reagents

The reagent grade chemicals and cell culture compounds used, namely Dulbecco’s Modified Eagle’s Medium (DMEM + GlutaMAX™), antibiotic solution (penicillin-streptomycin), non-essential amino acids (NEAAs), 4-(2-hydroxyethyl)-1-piperazineethanesulfonic acid (HEPES), fungizone, trypsin/EDTA solutions, Phosphate Buffer Saline (PBS), Fetal Bovine Serum (FBS), methylthiazoltetrazolium salt (MTT) dye and dimethyl sulfoxide (DMSO), were purchased from Sigma-Aldrich (St. Louis, MO, USA). Deionized water (resistivity <18 MW cm) was obtained by filtering tap water through a Milli-Q water purification system (Millipore, Bedford, MA, USA). Standards of vit C (176.12 g/mol), vit E (430.71 g/mol), QUE (338.27 g/mol) and RSV (228.24 g/mol) were purchased from Sigma-Aldrich (St. Louis, MO, USA). Protein hydrolyzates based on hydrolyzed salmon fish protein with added vitamin C and vitamin D3 (product 1), hydrolyzed salmon and mackerel fish protein with added vitamin C and vitamin D3 (product 2), hydrolyzed salmon and blue whiting fish protein with added vitamin C and vitamin D3 (product 3) and flounder skin collagen were kindly provided by NOFIMA (Norway). Stock solutions of antioxidants vit E, QUE and RSV were freshly prepared in DMSO and stock solution of vit C was prepared in H_2_O at appropriate working concentrations while maintained in darkness at 4 °C.

### 4.2. Cell Culture and Treatments

The Caco-2 cells were cultured in DMEM medium supplemented with 10% FBS, 1% HEPES, 1% NEAA, 0.2% fungizone, 100 U/mL penicillin and 100 mg/mL streptomycin. The incubation conditions were pH 7.4, 5% CO_2_ at 37 °C and 95% air atmosphere at constant humidity. The medium was changed every 2–3 days. Clonal line Caco-2 cells were kindly provided by the Central Service for Experimental Research (SCSIE) of the University of Valencia (Valencia, Spain).

### 4.3. Bioaccessibility and Bioavailability

To obtain the bioaccessible fraction, the standardized INFOGEST method was applied. Simulated Salivary Fluid (SSF), Simulated Gastric Fluid (SGF), Simulated Intestinal Fluid (SIF) and enzymatic activity assays were prepared according to Minekus et al. [41]. Briefly, 2.5 mL of each product or collagen solution, 2 mL of SSF, 12.5 µL of 0.3 M CaCl_2_ and deionized water to a final volume of 5 mL were mixed for 2 min. Afterwards, to simulate the gastric phase, 3.75 mL of SGF, 0.8 mL of pepsin solution (25,000 U/mL) and 2.5 µL of 0.3 M CaCl_2_ were added. The pH was then adjusted to 3.0, and deionized water was added up to a final volume of 10 mL. This gastric mixture was incubated for 2 h. Subsequently, 5.5 mL of SIF containing pancreatin (100 U trypsin activity/mL), bile salts (10 mmol/L) and 20 µL of 0.3 M CaCl_2_ was added. The pH was adjusted to 7.0, deionized water was added up to a final volume of 20 mL and the intestinal mixture was incubated for 2 h. The oral, gastric and intestinal steps were performed by mechanical shaking at 95 rpm and 37 °C. At the end of the in vitro digestive process, samples were cooled in an ice bath and centrifuged at 3100× *g* and 4 °C for 60 min to obtain the bioaccessible fraction.

The Caco-2 cells were seeded at a density of 2.25 × 10^5^ cells in inserts of 6-well Transwell Permeable Supports 12 mm in diameter (Corning Life sciences, Corning, NY, USA) at a pore size of 0.4 mm and were grown for 7 days. The culture medium in the apical and basolateral sides was replaced every 2–3 days. The integrity of the Caco-2 cell monolayers was confirmed by measuring transepithelial electrical resistance (TEER) using an electric resistance device (Millicell-ERS, Millipore Corp, Burlington, MA, USA), and monolayers with TEER above 300 W/cm^2^ were used for the bioavailability assay. The medium of the apical (upper compartment) and basolateral side (lower compartment) was removed, and transport was assessed by paracellular passage of the bioaccessible fraction of products or collagen (0.1 mg/mL) from the apical side to the basolateral side. Therefore, 1.5 mL of the bioaccessible fraction of digested hydrolyzates was added to the apical side and 2 mL of medium without serum was added to the basolateral side. Control samples comprising transport medium without serum were also evaluated. The medium of the basolateral side, which is considered the bioavailable fraction, was collected.

### 4.4. In Vitro Cytotoxicity Assay

The cytotoxic effects of samples were determined in Caco-2 cells by the MTT assay. The MTT assay determines the viability of cells through the reduction of yellow soluble MTT in metabolically active cells via a mitochondrial-dependent reaction to an insoluble purple formazan crystal. The MTT viability assay was performed according to Ruiz et al. [42].

Briefly, the Caco-2 cells were plated in 96-well tissue culture plates at a density of 2 × 10^4^ cells/well. After the cells reached 80% confluence, the culture medium was replaced with fresh medium containing serial dilutions of each product. The Caco-2 cells were exposed to serial dilutions of the bioaccessible fraction of products 1, 2 and 3 or collagen using fresh medium. The range of concentrations of the bioaccessible fractions were 1:2 (0.5 mg/mL), 1:4 (0.25 mg/mL), 1:10 (0.10 mg/mL) and 1:20 (0.05 mg/mL). The bioaccessible fractions were exposed in the Caco-2 cells over 24 h. During the exposure time, neither the medium nor the bioaccessible fraction was replenished. After 24 h of exposure, the medium was removed and 200 µL of fresh medium was added to each well. Subsequently, 50 µL/well of MTT was added, and the plates were returned to the incubator in the dark. After 3 h of incubation, the MTT solution was removed and 200 µL of DMSO was added, followed by 25 µL of Sorensen’s glycine buffer. Plates were gently shaken for 5 min to achieve complete dissolution. Absorbance was measured at 540 nm using an automatic ELISA plate reader (MultiSkanEX, Thermo Scientific, Waltham, MA, USA).

In parallel, pre-treatment with antioxidants was conducted in Caco-2 cells, which exposed the bioaccessible fraction of these compounds. Antioxidants (vit C, vit E, QUE and RSV) were combined with 0.1 and 0.2 mg/mL of each bioaccessible fraction of each product and collagen. The selected concentrations were 200 µM and 400 µM for vit C, 10 µM and 25 µM for vit E and 5 µM and 10 µM for QUE and RSV. The concentrations assayed were selected considering two criteria: the nutritional reference intake of antioxidants and the concentrations selected in previous studies. The selected concentrations of bioaccessible fractions (0.1 and 0.2 mg/mL) were chosen because cell viability started to decrease at these concentrations in Caco-2 cells during the MTT assay. The results obtained in these assays were compared with the cell viability results obtained with the bioaccessible fraction without antioxidant pre-treatment.

For the MTT assay, cell viability was expressed as a percentage relative to the control solvent (medium DMEM + GlutaMAX™). Determinations were performed in three independent experiments with 4 replicates for each one. The mean inhibition concentration (IC_50_) values were calculated using SigmaPlot version 11 (Systat Software Inc., GmbH, Düsseldorf, Germany).

### 4.5. Analysis of Minerals in Fish Products

The presence and content of Mg, P, Ca, Fe, Zn and Se in products 1, 2 and 3 and collagen was evaluated. A microwave-accelerated reaction system (MARS, CEM, Vertex, Madrid, Spain) was used for the acid mineralization of samples. Briefly, in pre- and post-simulated gastrointestinal digestion samples, 0.1–0.5 mL of sample was placed in a Teflon vessel (MARS, CEM, Vertex, Madrid, Spain). Subsequently, 0.5 mL of HNO_3_ (69% *v*/*v*) was added to the samples and digestion was carried out in the microwave system at 800 W and 180 °C for 15 min. After cooling and eliminating the nitrogen vapors, the digested samples were filtered through Whatman No. 1 filter paper and total volume was brought up to 5 mL with distilled water. In samples of bioavailable fractions, 0.75 mL of sample was placed in a Teflon vessel. Following this, 1 mL of HNO_3_ (69% *v*/*v*) was added to the samples and digestion was carried out in the microwave system at 800 W and 180 °C for 15 min. After cooling and eliminating the nitrogen vapors, the digested samples were filtered through Whatman No. 1 filter paper and total volume was brought up to 5 mL with distilled water. For all samples, an inductively coupled plasma spectrometer mass detector (ICP MS, Agilent model 7900, Santa Clara, CA, USA) was then employed to identify and quantify the metals. The operating conditions were as follows: Ar plasma gas flow, 15.0 L/min; carrier gas, 1.0 L/min; reaction gas, He; nebulizer pump speed, 0.30 rps; RF power, 1550 W; RF matching, 1.80 V. Internal standard solutions of ^45^Sc and ^72^Ge (ISC Science, Paris, France) at 20 µg/g were used to correct signal fluctuations and instrumental drift. This mineral analysis technique was used previously by Calleja-Gómez et al. [43].

A standard calibration curve with concentrations ranging from 0 to 10,000 µg/mL was used for the quantification of Mg, P, Ca, Fe, Zn and Se. Distilled water was used as a blank and the metal concentrations in the digested blank were subtracted from the sample values. In pre- and post-simulated gastrointestinal digestion samples, the results were expressed as mg of each element/L of fish product in wet weight, and in samples of bioavailable fractions, the results were expressed as mg of each element/Kg of fish product in wet weight.

### 4.6. Analysis of Metals in Fish Products

The presence and content of As, Hg, Cd and Pb in products 1, 2 and 3 and collagen was evaluated. A microwave-accelerated reaction system (MARS, CEM, Vertex, Spain) was used for the acid mineralization of samples. Briefly, in pre-simulated gastrointestinal digestion samples, 0.5 mL of sample was placed in a Teflon vessel. Subsequently, 0.5 mL of HNO_3_ (69% *v*/*v*) was added to the samples and digestion was carried out in the microwave system at 800 W and 180 °C for 15 min. After cooling and eliminating the nitrogen vapors, the digested samples were filtered through Whatman No. 1 filter paper and total volume was brought up to 5 mL with distilled water. In samples of bioavailable fractions, 0.75 mL of sample was placed in a Teflon vessel. Following this, 1 mL of HNO_3_ (69% *v*/*v*) was added to the samples and digestion was carried out in the microwave system at 800 W and 180 °C for 15 min. After cooling and eliminating the nitrogen vapors, the digested samples were filtered through Whatman No. 1 filter paper and total volume was brought up to 5 mL with distilled water. For all samples, an inductively coupled plasma spectrometer mass detector (ICP MS, Agilent model 7900) was then employed to identify and quantify the metals. The operating conditions were as follows: Ar plasma gas flow, 15.0 L/min; carrier gas, 1.0 L/min; reaction gas, He; nebulizer pump speed, 0.30 rps; RF power, 1550 W; RF matching, 1.80 V. Internal standard solutions of ^72^Ge, ^103^Rh and ^193^Ir (ISC Science) at 20 µg/g were used to correct signal fluctuations and instrumental drift. This metals analysis technique was used previously by Taroncher et al. [44].

A standard calibration curve with concentrations ranging from 0 to 10,000 µg/L was used for the quantification of As, Cd, Hg and Pb. Distilled water was used as a blank and the metal concentrations in the digested blank were subtracted from the sample values. In pre-simulated gastrointestinal digestion samples, the results were expressed as µg of each element/L of fish product in wet weight, and in samples of bioavailable fractions, the results were expressed as mg of each element/Kg of fish product in wet weight.

### 4.7. Statistical Analysis

Statistical analysis of the data was carried out using the Statgraphics version 16.01.03 statistical package (IBM Corp., Armonk, NY, USA). Data were expressed as the mean ± standard error of the mean (SEM) of different independent experiments. Statistical analysis of the results was performed using the Student *t*-test for paired samples. Differences between groups were analyzed using one-way analysis of variance (ANOVA) followed by Tukey’s HDS post hoc test for multiple comparisons. Statistical significance was considered as *p* ≤ 0.05.

## 5. Conclusions

In conclusion, the results of this study show that the studied natural antioxidants (vitamins C and E, QUE and RSV) enhanced the cell proliferation of fish protein hydrolyzates in Caco-2 cells, suggesting that consumption of these antioxidants with the hydrolyzates would be necessary to produce less cell damage. However, more studies are needed to fully understand the health properties of discarded fish products and realize the maximum benefits for industries, the environment and consumers.

## Figures and Tables

**Table 1:** Mineral content before simulated gastrointestinal digestion of product 1, product 2, product 3 and collagen.

Sample	Mg (mg/L)	P (mg/L)	Ca (mg/L)	Fe (mg/L)	Zn (mg/L)	Se (mg/L)
Product 1	76.0 ± 1.5	510.0 ± 4.0	65.0 ± 1.7	0.544 ± 0.006	2.65 ± 0.020	0.140 ± 0.008
Product 2	218.0 ± 3.0	366.0 ± 8.0	85.8 ± 1.4	0.338 ± 0.006	0.64 ± 0.011	0.720 ± 0.030
Product 3	123.0 ± 2.0	565.0 ± 18.0	50.0 ± 0.6	0.445 ± 0.007	1.40 ± 0.002	0.193 ± 0.018
Collagen	78.3 ± 1.8	25.5 ± 0.8	633.0 ± 12.0	0.205 ± 0.001	0.12 ± 0.001	0.049 ± 0.003

**Table 2:** Mineral content after simulated gastrointestinal digestion of product 1, product 2, product 3 and collagen.

Sample	Mg (mg/L)	P (mg/L)	Ca (mg/L)	Fe (mg/L)	Zn (mg/L)	Se (mg/L)
Product 1	9.1 ± 0.4	46.0 ± 1.0	7.9 ± 1.2	0.057 ± 0.007	0.190 ± 0.030	<0.020
Product 2	30.1 ± 0.6	7.4 ± 1.8	17.7 ± 0.7	<0.000	<0.000	0.110 ± 0.009
Product 3	16.6 ± 0.5	52.6 ± 3.0	16.7 ± 0.8	<0.000	<0.000	0.034 ± 0.003
Collagen	7.7 ± 0.4	<0.0	59.1 ± 2.0	<0.000	<0.000	<0.020

**Table 3:** Mineral content of the bioavailable fraction of product 1, product 2, product 3 and collagen.

Sample	Mg (mg/Kg)	P (mg/Kg)	Ca (mg/Kg)	Fe (μg/Kg)	Zn (μg/Kg)	Se (μg/Kg)
Product 1	<0.0	0.7 ± 0.7	<0.0	<0.0	65.3 ± 0.7	<1.0
Product 2	<0.0	<0.0	<0.0	<0.0	14.0 ± 3.0	3.9 ± 0.2
Product 3	<0.0	0.1 ± 0.7	<0.0	<0.0	<0.0	<1.0
Collagen	<0.0	1.6 ± 0.6	<0.0	<0.0	0.0 ± 2.0	<1.0

**Table 4:** Metal content before simulated gastrointestinal digestion of product 1, product 2, product 3 and collagen.

Sample	As (μg/L)	Cd (μg/L)	Hg (μg/L)	Pb (μg/L)
Product 1	1149.00 ± 30.00	1.20 ± 0.14	4.50 ± 0.30	2.06 ± 0.07
Product 2	307.00 ± 8.00	16.50 ± 1.10	0.90 ± 0.01	1.19 ± 0.07
Product 3	1421.00 ± 15.00	2.35 ± 0.18	3.30 ± 0.20	1.10 ± 0.03
Collagen	72.60 ± 0.90	<0.20	0.13 ± 0.02	1.25 ± 0.05

**Table 5:** Metal content of the bioavailable fraction of product 1, product 2, product 3 and collagen.

Sample	As (μg/Kg)	Cd (μg/Kg)	Hg (μg/Kg)	Pb (μg/Kg)
Basal product 1	0.41 ± 0.03	<0.20	<0.00	<0.00
Basal product 2	<0.25	<0.20	<0.00	<0.00
Basal product 3	<0.25	<0.20	0.18 ± 0.07	<0.00
Basal collagen	<0.25	<0.20	<0.00	<0.00

## Data Availability

Data are contained within the article.
